# Inhibition of phosphodiesterase 5 reduces bone mass by suppression of canonical Wnt signaling

**DOI:** 10.1038/cddis.2014.510

**Published:** 2014-11-27

**Authors:** Y Gong, C Y Xu, J R Wang, X H Hu, D Hong, X Ji, W Shi, H X Chen, H B Wang, X M Wu

**Affiliations:** 1 Department of Pharmacology, School of Medicine, Zhejiang University, Hangzhou 310058, China; 2 Department of Orthopedics, Taizhou Hospital, Linhai 317000, China; 3 State Key Laboratory of Reproductive Biology, Institute of Zoology, Chinese Academy of Sciences, Beijing 100101, China

## Abstract

Inhibitors of phosphodiesterase 5 (PDE5) are widely used to treat erectile dysfunction and pulmonary hypertension in clinics. PDE5, cyclic guanosine monophosphate (cGMP), and protein kinase G (PKG) are important components of the non-canonical Wnt signaling. This study aimed to investigate the effect of PDE5 inhibition on canonical Wnt signaling and osteoblastogenesis, using both *in vitro* cell culture and *in vivo* animal models. In the *in vitro* experiments, PDE5 inhibition resulted in activation of cGMP-dependent protein kinase 2 and consequent inhibition of glycogen synthase kinase 3*β* phosphorylation, destabilization of cytosolic *β*-catenin and the ultimate suppression of canonical Wnt signaling and reduced osteoblastic differentiation in HEK293T and C3H10T1/2 cells. In animal experiments, systemic inhibition of PDE5 suppressed the activity of canonical Wnt signaling and osteoblastogenesis in bone marrow-derived stromal cells, resulting in the reduction of bone mass in wild-type adult C57B/6 mice, significantly attenuated secreted Frizzled-related protein-1 (SFRP1) deletion-induced activation of canonical Wnt signaling and excessive bone growth in adult SFRP1^−/−^ mice. Together, these results uncover a hitherto uncharacterized role of PDE5/cGMP/PKG signaling in bone homeostasis and provide the evidence that long-term treatment with PDE5 inhibitors at a high dosage may potentially cause bone catabolism.

In the canonical Wnt (Wnt/*β*-catenin (*β*-cat)) signaling cascade, Wnt binds to Frizzled (Frz) receptors and the low-density lipoprotein receptor-related protein (LRP) 5 or 6, thereby activating dishevelled, suppressing the glycogen synthase kinase 3*β* (GSK3*β*) activity and inhibiting phosphorylation of *β*-cat at Thr41, Ser37, and Ser33 sites. The stabilized cytosolic *β*-cat enters the nucleus and consequently activates its downstream target genes via lymphoid enhancer-binding factor-1 (Lef-1) and T-cell factors.^1, 2^ This signaling is fine-tuned in part via a negative feedback mechanism involving secreted and transmembrane Wnt inhibitors and activators, secreted Frz-related proteins (SFRPs), and Dickkopf-1 (Dkk1).^3, 4^

Canonical Wnt signaling is critical not only to bone development in embryogenesis but also to the maintenance of bone mass during adult life.^5^ The initial evidence came from the discoveries that in humans loss- or gain-of-function mutations in LRP5 were linked with the osteoporosis-pseudoglioma syndrome and a high-bone-density syndrome, respectively.^6, 7, 8^ Subsequent studies in mice showed that Wnt signaling might promote ossification by inducing the differentiation of bone-forming osteoblasts, suppressing the development of bone-resorbing osteoclasts, and driving the differentiation of multi-potent stem cells toward an osteoblast cell fate.^9^

Non-canonical Wnt signaling is *β*-cat independent and consists of two main pathways: the Rho small GTPases-mediated planar cell polarity pathway and the Wnt/Ca^2+^ pathway,^10^ involved in various aspects of cell fate differentiation and cell movement. Non-canonical Wnt signaling has profound effects on tissue morphogenesis in a variety of vertebrate species.^10^ The potential role for non-canonical Wnt signaling in bone formation has been investigated recently in limited studies, which have shown that the non-canonical Wnt-Gαq/11-PKC pathway operates in mammalian osteoprogenitors to promote osteoblast development, and that Wnt16 exhibits a stimulatory effect on bone metabolism.^11, 12, 13^ Nevertheless, the molecular events in the non-canonical Wnt signaling regulation of bone development and homeostasis have yet to be further elucidated.

Phosphodiesterases (PDEs) are a large family of enzymes that cleave cyclic nucleotides. To date, 11 PDE subtypes have been identified, among which PDE5 has been most extensively studied. PDE5, cyclic guanosine monophosphate (cGMP), and cGMP-dependent protein kinase (PKG) are among the major components of the non-canonical Wnt signaling pathway and are involved in the regulation of intracellular Ca^2+^ concentration.^14, 15^ It is now well established that PDE5 degrades 3'-5′- cGMP and its inhibition leads to an increase in intracellular cGMP levels and activation of protein kinase G (PKG), resulting in a decrease in Ca^2+^ influx and consequent relaxation of smooth muscles, which produces the therapeutic effects in clinical erectile dysfunction (ED) and pulmonary hypertension (PH).^14^ Currently, little is known regarding the involvement of PDE5 in Wnt signaling regulation of bone formation and homeostasis. The objective of this study was to determine the effect of PDE5 inhibition on canonical Wnt signaling and bone mass.

## Results

### Involvement of PDE5 and PKG in canonical Wnt signaling

**Figure 1:**
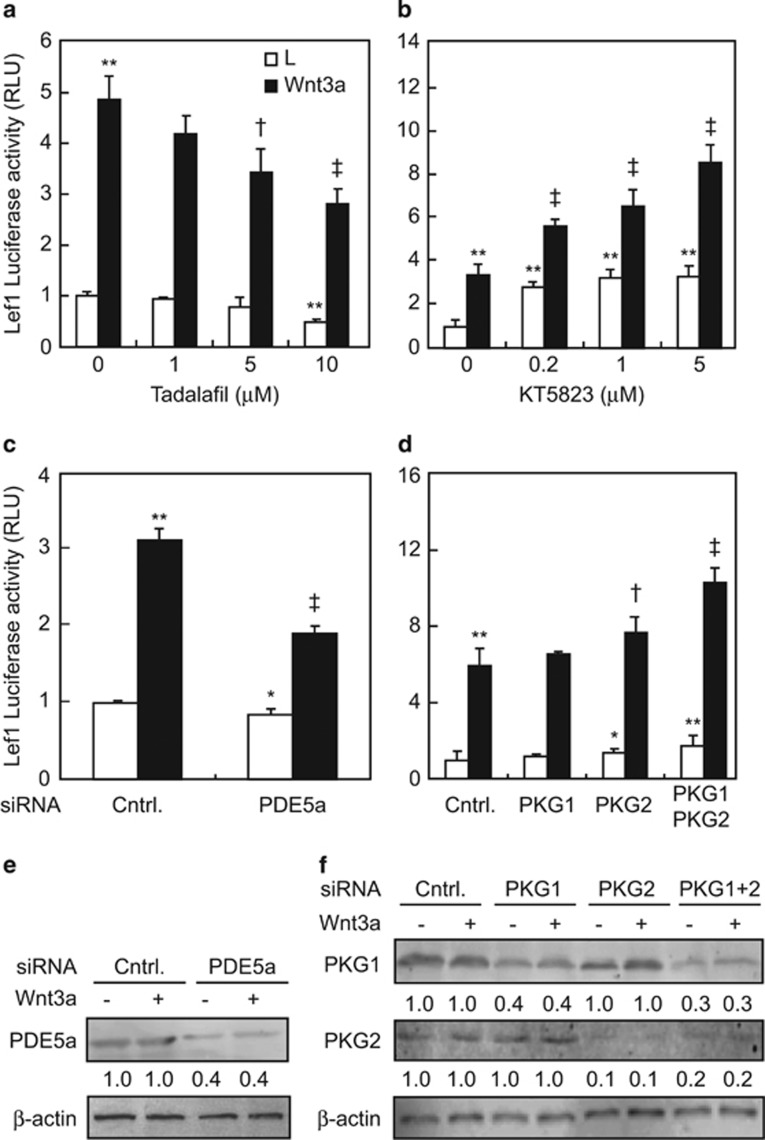
PDE5 and PKG2 regulates canonical Wnt signaling in 293T cells. (**a** and **b**) PDE5 inhibitor tadalafil and PKG inhibitor KT5823 affect the Lef1 luciferase activity. After co-transfection with the Lef1 luciferase and Renilla luciferase plasmids, 293T cells were treated with indicated concentrations of either tadalafil or KT5823 for 24 h in the presence of either control L cell-conditioned medium (L) or Wnt3a-expressing cell-conditioned medium (Wnt3a). (**c** and **d**) PDE5a, PKG1, and PKG2 siRNAs affect the Lef1 luciferase activity. After co-transfection with the reporter constructs and respective siRNAs, 293T cells were further cultured for 24 h in the presence of L(−) or Wnt3a (+) conditional medium. (**e** and **f**) Protein levels of PDE5a, PKG1, and PKG2 in cells transfected with siRNAs. The signal form first band was defined as 1. *P<0.05, **P<0.01 *versus* L medium and vehicle treatments (or control siRNA); ^†^*P*<0.05, ^‡^*P*<0.01 *versus* Wnt3a medium and vehicle treatments (or control siRNA)

To examine the possibility of PDE5 and PKG in canonical Wnt signaling, we determined the effects of PDE5 and PKG inhibitors, and PDE5- and PKG-specific siRNAs on Lef1 reporter activity in 293 T cells. The two PDE5 inhibitors (i.e., tadalafil and vardenafil) used inhibited the Lef1-luciferase activity in a dose-dependent manner; at 10 *μ*M, tadalafil and vardenafil decreased the reporter activity by 58 and 30%, respectively, in the presence of control L cell-conditioned medium (L), and by 42 and 60%, respectively, in the presence of Wnt3a-expressing cell-conditioned medium (Wnt3a) (Figure 1a and Supplementary Figure 1a). In contrast, KT5823, a potent inhibitor of PKG, dose dependently induced the Lef1-luciferase activity in 293 T cells; at 5 *μ*M, it increased the reporter activity by 2.4- and 1.5-fold, respectively, in the presence of control L cell-conditioned medium and Wnt3a-expressing cell-conditioned medium (Figure 1b). Neither tadalafil nor KT5823 at the indicated concentrations affected the apoptosis of either 293 T cells or C3H10T1/2 cells, after 24 h treatments (Supplementary Figure 1c–e). To further confirm the specificity of PDE5 and PKG inhibitors in regulation of canonical Wnt signaling, we transfected the cells with either PDE5a or PKG siRNA and determined the Lef1 reporter activities. Wnt3a affected neither the cGMP levels nor the PDE5a expression (Figure 1e and Supplementary Figure 1b). PDE5a siRNA decreased PDE5a expression by ~60% at the protein level and suppressed Lef1-luciferase activity by 15 and 45% in the presence of control L cell-conditioned medium and Wnt3a-expressing cell-conditioned medium, respectively (Figures 1c and e). PKG1 and PKG2 siRNAs, each lone, reduced PKG1 and PKG2 protein contents by 60 and 90%, respectively, whereas the combination of PKG1 and PKG2 siRNAs reduced PKG1 and PKG2 protein contents by 70 and 80%, respectively (Figure 1f). Silencing of PKG2 but not PKG1 resulted in a decrease in Lef1-luciferase activities by 20 and 33% in the presence of control L cell-conditioned medium and Wnt3a-expressing cell-conditioned medium, respectively, and knockdown of both PKG1 and PKG2 decreased Lef1-luciferase activities by ~70 and 75% in the presence of the two conditioned media, respectively (Figure 1d). Thus, inhibition of PDE5 negates canonical Wnt signaling, in line with this observation, and suppression of PKG, especially PKG2, induces canonical Wnt signaling in either the presence or absence of Wnt3a.

### Effects of PDE5 inhibition on *β*-cat expression and stabilization

**Figure 2:**
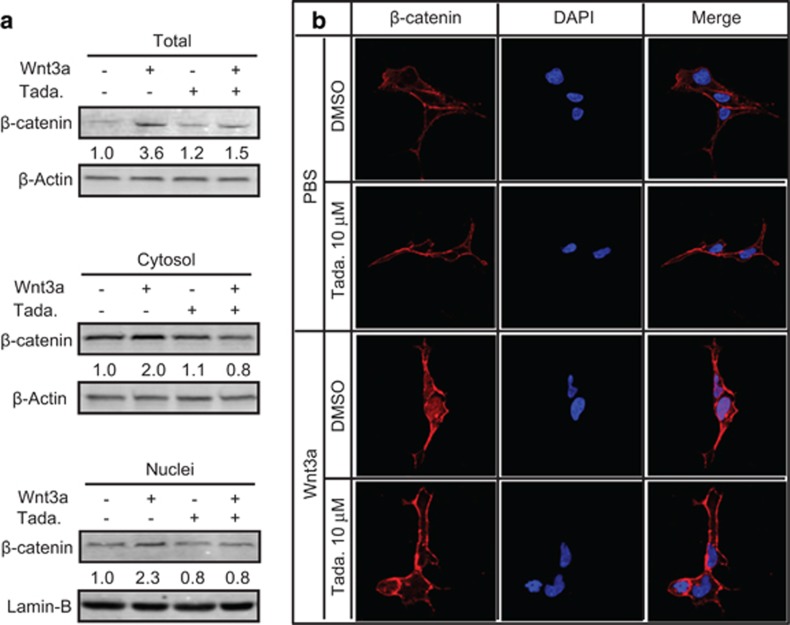
*β*-Catenin levels in 293T cells treated with PDE5 inhibitor in the presence or absence of Wnt3a. (**a**) Tadalafil reduced *β*-catenin protein levels. Western blot analyses of *β*-catenin levels in whole cells, cytosolic, and nuclear fractions of 293T cells treated with vehicle (−) or tadalafil (+) at 10 *μ*M in the presence of L(−) or Wnt3a (+) conditional medium for 24 h. (**b**) Immunofluorescence and confocal images of 293T cells treated with dimethyl sulfoxide (DMSO) or tadalafil at 10 *μ*M in the presence or absence of recombinant Wnt3a for 24 h

Given that *β*-cat is the central signal transducer of the Wnt signaling pathway, we evaluated the effect of PDE5 inhibition on *β*-cat messenger RNA (mRNA) abundance and protein content, and stabilization in 293 T cells, to further assess the possible involvement of PDE5 in Wnt signaling. PDE5 inhibitor tadalafil did not significantly change *β*-cat mRNA levels at either 5 or 10 *μ*M but attenuated Wnt3a-induced increases in *β*-cat protein levels in whole-cell lysates, cytosolic, and nuclear fractions by 58, 60, and 65%, respectively, at 10 *μ*M (Supplementary Figure 2a and Figure 2a). Immunostaining further confirmed the effect of tadalafil on *β*-cat protein content; at 10 *μ*M, tadalafil decreased both cytosolic and nuclear *β*-cat protein levels in response to recombinant Wnt3a protein (Figure 2b). Thus, inhibition of PDE5 negates canonical Wnt signaling through decreasing *β*-cat levels.

**Figure 3:**
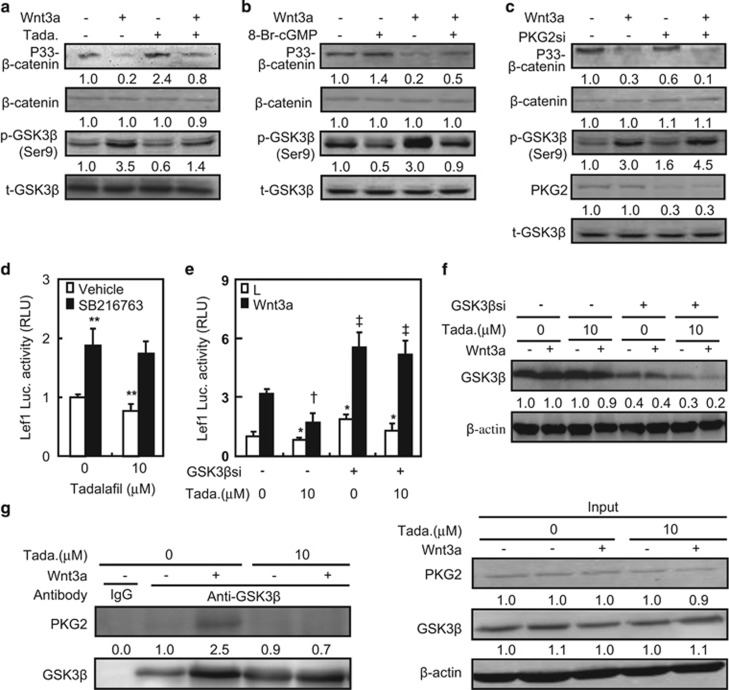
Stabilization of *β*-catenin affected by PDE5/cGMP/PKG2 signals in 293T cells. (**a** and **b**) Tadalafil and 8-Br-cGMP affected the activation of GSK3*β* and stabilization of *β*-catenin. 293T cells were pretreated with vehicle (−) or tadalafil (+) at 10 *μ*M for 3 h, then were stimulated with L(−) or Wnt3a (+) medium in the presence of vehicle or tadalafil (10 *μ*M) for further 30 min. p33-*β*-catenin, total *β*-catenin, p-GSK3*β* (Ser9), and total GSK3*β* were measured by western blottings. (**c**) PKG2 siRNA inhibited the activation of GSK3*β* and stabilization of *β*-catenin. 293T cells were transfected with siRNA for 48 h, then were stimulated with L or Wnt3a medium in the presence of vehicle or tadalafil (10 *μ*M) for further 30 min. (**d** and **e**) Effects of GSK3*β* inhibitor or its siRNA on Lef1 luciferase activities in 293T cells treated with or without tadalafil in the presence of L or Wnt3a conditional medium. (**f**) Protein levels of GSK3*β* in 293T cells transfected with its siRNAs. (**g**) Co-immunoprecipitation of endogenous GSK3*β* and PKG2. After various treatments, 293T cells were subjected to immunoprecipitation and western blot analyses by using the indicated antibodies. The signal from the first band was defined as 1. *P<0.05, **P<0.01 *versus* vehicle treatment and in the presence or absence of L medium and control siRNA; ^†^*P*<0.05, ^‡^*P*<0.01 *versus* Wnt3a medium, vehicle, and control siRNA treatments

Phosphorylation of *β*-cat at Ser45 by casein kinase-1 (CK1) and at Thr41, Ser37, and Ser33 by GSK3*β* regulates its stabilization in the cytosol, and the phosphorylated *β*-cat is recognized by E3 ubiquitin ligase and undergoes proteolytic degradation.^16^ To determine the role of PDE5 in the stabilization of *β*-cat, we assessed changes in *β*-cat phosphorylation in 293T cells treated with tadalafil at 10 *μ*M, in either the presence or absence of Wnt3a. Wnt3a slightly decreased the phosphorylation of *β*-cat at Ser45, whereas tadalafil showed no significant effect (Supplementary Figure 2b). Phosphorylation of *β*-cat at Ser33, Ser37, and Thr41 was reduced by Wnt3a (80%) but induced by tadalafil (1.4-fold), and tadalafil was capable of reversing the negative effect of Wnt3a on *β*-cat phosphorylation (3-fold) (Figure 3a). In contrast, Wnt3a increased (2.5-fold) but tadalafil decreased (40%) GSK3*β* phosphorylation at Ser9, and tadalafil was effective to attenuate the Wnt3a-induced GSK3*β* phosphorylation (60%) (Figure 3a).

We also assessed the possible involvement of cGMP in the destabilization of *β*-cat. We observed that the cGMP analog, 8-Br-cGMP, increased *β*-cat phosphorylation by 40% at Thr41, Ser37, and Ser33, but decreased GSK3*β* phosphorylation by 50% at Ser9 in 293T cells (Figure 3b). Conversely, Wnt3a resulted in a decrease in *β*-cat phosphorylation (80%) but an increase in GSK3*β* phosphorylation (2-fold) (Figure 3b). These effects of Wnt3a were robustly reversed by 8-Br-cGMP (1.5-fold for *β*-cat and 70% for GSK3*β*) (Figure 3b). We further evaluated the potential role for PKG2 in *β*-cat stabilization using the siRNA technology. PKG2-specific siRNA, PKG2si, reduced the expression of PKG2 by 70% and decreased *β*-cat phosphorylation by 40%, but increased GSK3*β* phosphorylation by 60% (Figure 3c). Moreover, PKG2si synergistically attenuated Wnt3a's inhibitory effect on *β*-cat phosphorylation (66%) and potentiated Wnt3a's induction of GSK3*β* phosphorylation (50%) (Figure 3c).

Finally, we performed reporter assays and co-immunoprecipitation to confirm GSK3*β* signaling as a downstream event of PDE5/cGMP/PKG2 signals in the regulation of *β*-cat stabilization. GSK3*β* inhibitor, SB216763, increased Lef1-luciferase activity but showed no significant effect in the presence of tadalafil in 293T cells (Figure 3d). However, the presence of tadalafil had no effect on Wnt3a induction of Lef1-luciferase activity in 293T cells expressing GSK3*β* siRNA (GSK3*β*si) where GSK3*β* protein content was decreased by 60~80% (Figures 3e and f). In cells treated with Wnt3a alone, protein complex precipitated with a GSK3*β* antibody contained a considerable amount of PKG2; however, in cells treated with other test agents, protein complex contained a small amount of PKG2 in addition to GSK3*β*, as expected (Figure 3g). Taken together, these observations suggest that activation of PKG2 by inhibition of PDE5 may suppress canonical Wnt signaling through inducing GSK3*β*-mediated phosphorylation of *β*-cat at Thr41, Ser37, and Ser33 sites.

### Effects of PDE5 inhibition on osteoblastic differentiation

**Figure 4:**
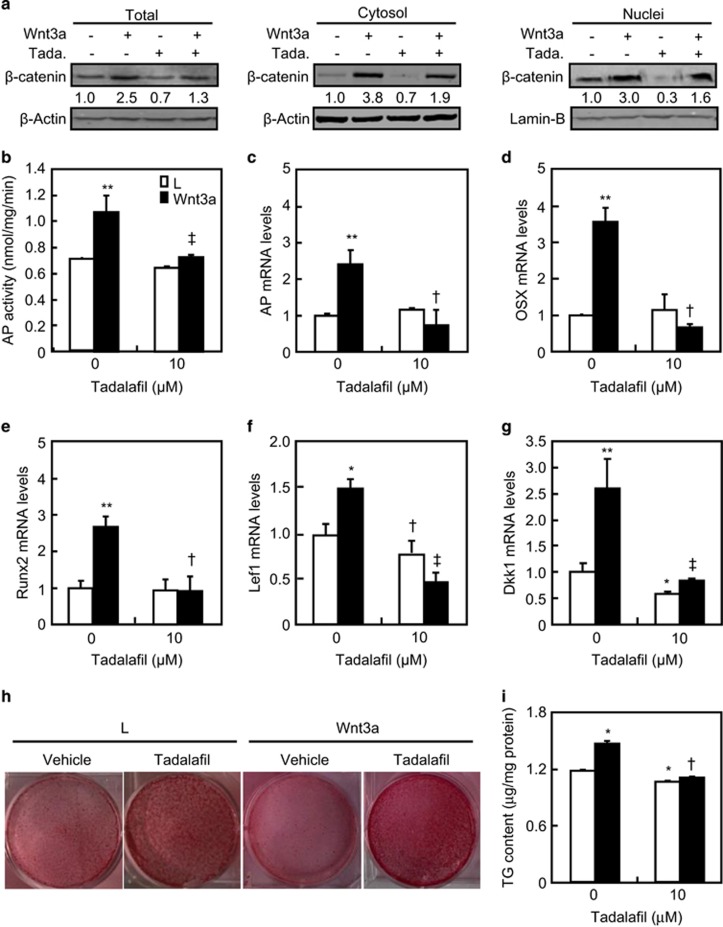
Inhibition of PDE5 suppresses the differentiation of osteoblast. (**a**) Tadalafil reduced *β*-catenin protein levels. Western blot analyses of *β*-catenin levels in whole cells, cytosolic, and nuclear fractions of C3H10T1/2 cells treated with vehicle (−) or tadalafil (+) at 10 *μ*M in the presence of L(−) or Wnt3a (+) conditional medium for 24 h. (**b**–**g**) Expression of osteoblast differential markers and target gene of canonical Wnt signaling in C3H10T1/2 cells treated with tadalafil in the presence of L or Wnt3a medium. Cells were cultured in L or Wnt3a medium in the presence of vehicle or tadalafil at 10 *μ*M for 48 h followed by AP activity analyses and quantitative RT-PCR assays for mRNA levels of AP, OSX, Runx2, Lef, and Dkk1. (**h** and **i**) Tadalafil reduced the formation of mineralized nodules in response to Wnt3a medium. Cells were cultured in L or Wnt3a medium in the presence of ascorbic acid, *β*-glycerophosphate, and dexamethasone. After incubation for 21 days, cells were detected for bone nodules by alizarin-red staining and quantitative determination. The signal for the first western band was defined as 1. *P<0.05, **P<0.01 *versus* L medium and vehicle treatments. ^†^*P*<0.05, ^‡^*P*<0.01 *versus* Wnt3a medium and vehicle treatments

To examine the role of PDE5 in osteoblastogenesis, we tested the effect of tadalafil on *β*-cat expression and osteoblastic differentiation in the presence or absence of Wnt3a in embryonic fibroblast C3H10T1/2 cells. Similar to what was observed in 293T cells, tadalafil at 10 *μ*M attenuated Wnt3a-induced increases in *β*-cat protein content in the whole-cell lysate, cystosolic, and nuclear by 48, 50, and 47%, respectively, in C3H10T1/2 cells (Figure 4a). Moreover, Wnt3a not only increased mRNA levels of canonical Wnt signaling target genes including *Lef1* and *Dkk1* (Figures 4f and g) but also increased the differentiation of embryonic fibroblasts to osteoblasts as demonstrated by increases in mRNA levels of osteoblastogenic markers including alkaline phosphatase (AP), ostrix (OSX), and Runt-related transcription factor 2 (Runx2), as well as AP activities and mineralized nodule formation (Figures 4b–i). Although tadalafil alone affected neither mRNA levels of AP, OSX, and Runx2 nor AP activities, it slightly reduced the baseline mRNA levels of Lef1 and Dkk1, and the mineralized nodule formation (Figures 4b–i). In contrast, tadalafil reduced not only Wnt3a-induced increases in mRNA levels of Lef1 (72%) and DKK1 (75%) but also of osteoblastic markers including AP (74%), OSX (86%), and Runx2 (67%), and AP activities (33%) and formation of mineralized nodules (30%) (Figures 4b–i). Thus, in C3H10T1/2 cells, inhibition of PDE5 suppresses not only the canonical Wnt signaling but also the osteoblastogenesis in response to Wnt3a.

### Decreases in bone mass after systemic inhibition of PDE5 *in vivo*

**Figure 5:**
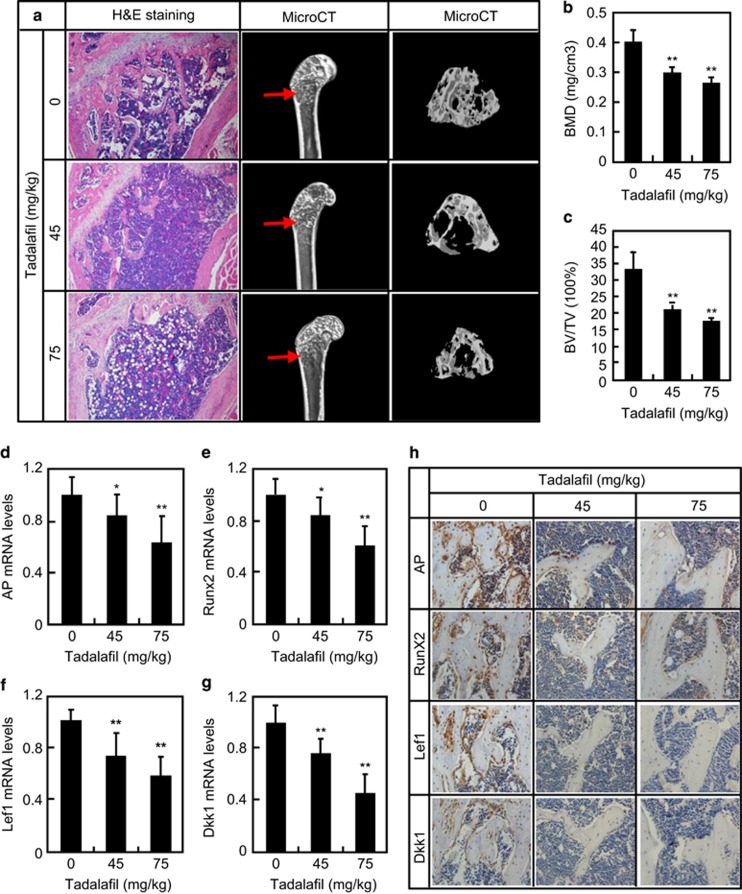
Tadalafil reduced bone mass in the adult wild-type mice. (**a**) Tadalafil reduced bone mass of distal femur in the adult mice. H&E staining of paraffin sections and *μ*CT analyses of the distal femur from mice intragastrically treated with normal saline or indicated dosages of tadalafil daily for 2 months. (**b** and **c**) Quantification of bone parameters from three-dimensional reconstruction *μ*CT. (**d**–**g**) Tadalafil treatment reduced the mRNA levels of osteoblast marker genes (*AP* and *RunX2*) and target genes of canonical Wnt signaling (*Lef1* and *Dkk1*) in BMSCs compared with the vehicle treatment. (**h**) Immunohistochemistry analyses of AP, Runx2, Lef1, and DKK1 expression in the distal femur of mice treated with vehicle or indicated dosages of tadalafil. *P<0.05, **P<0.01 *versus* vehicle treatment.

To assess the potential role of inhibition of PDE5 in bone mass *in vivo*, we first evaluated the effect of tadalafil on osteoblastogenesis and Wnt/*β*-cat signaling in wild-type adult (2 months) C57BL/6 mice. Orally administered for 2 months at 45 or75 mg/kg daily that was 20- and 32-fold higher than the clinical dosage for ED (10 mg daily) and 5- and 8-fold higher than the clinical dosage for PH (40 mg daily), ^17, 18^ tadalafil robustly decreased the mass of cancellous bone but not of cortical bone as revealed by morphological and histological analyses (Figure 5a). Three-dimensional reconstruction of the distal femur using micro computed tomography (*μ*CT) confirmed that tadalafil at both 45 and 75 mg/kg resulted in decreases in bone mineral density (BMD) by 30 and 35%, in trabecular bone volume (BMTV) by 42 and 48%, in trabecular number (TbN) by 35 and 50%, but increases in trabecular separation (TbSp) by 58 and 97%, respectively, while it had no effect on trabecular thickness (TbTh), when compared with the vehicle treatment (Figures 5a–c, and Supplementary Figure 3a and b). Moreover, tadalafil at 45 and 75 mg/kg reduced mRNA levels of Lef1 (34 and 49%, respectively) and Dkk1 (24 and 56%, respectively) in bone marrow-derived stromal cells (BMSCs) (Figures 5f and g). In line with the inhibition of canonical signaling, tadalafil dose dependently reduced mRNA levels of osteoblastogenic markers including AP (18 and 38%, respectively) and Runx2 (18 and 43%, respectively) (Figures 5d and e). Finally, tadalafil robustly decreased the numbers of not only Lef- and Dkk1-positive cells but also AP- and Runx2-positive cells as revealed by immunohistochemical staining of longitudinal sections of the distal femur (Figure 5h). Taken together, these results demonstrate that systemic inhibition of PDE5 may lead to robust inhibition of osteoblastogenesis and consequent reduction in bone mass possibly through inhibition of canonical Wnt signaling in adult mice.

**Figure 6:**
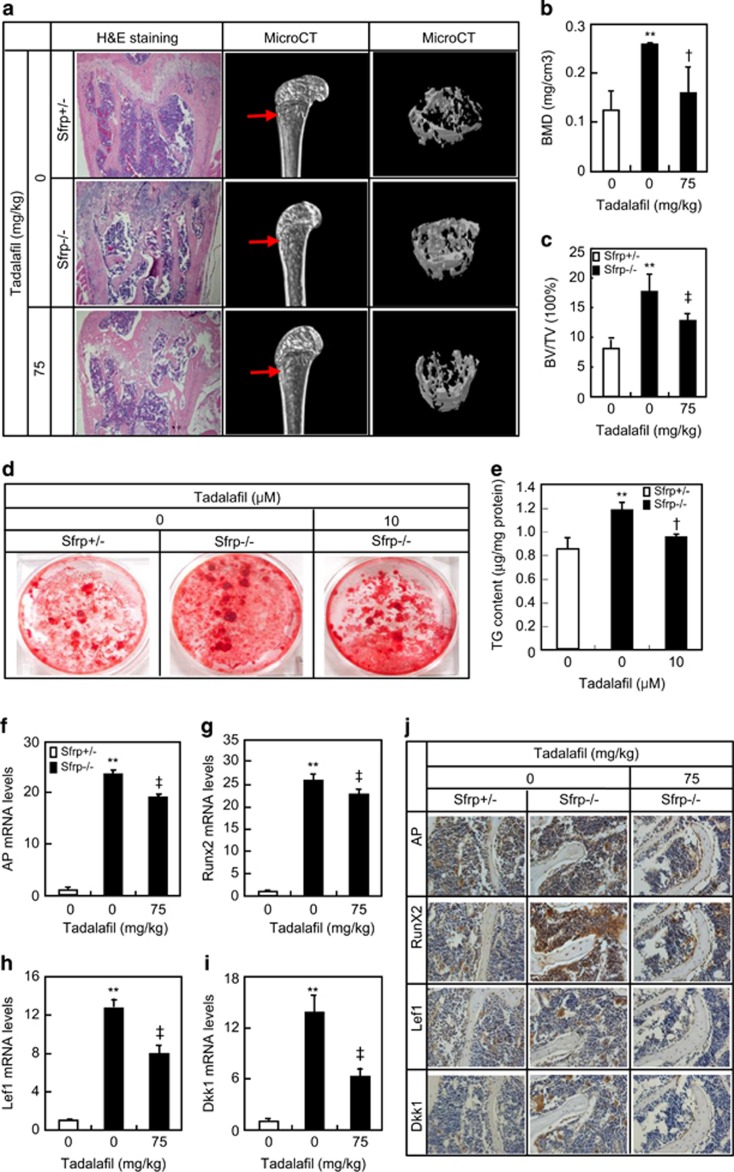
Tadalafil reduced bone mass in the adult SFRP1^−/−^ mice. (**a**) Tadalafil reduced bone mass of the distal femur in the adult SFRP1^−/−^ mice. H&E staining of paraffin sections and *μ*CT analyses of the distal femur from SFRP1^+/−^ or SFRP1^−/−^ mice intragastrically administrated with normal saline or indicated dosage of tadalafil daily for 2 months. (**b** and **c**) Quantification of bone parameters from three-dimensional reconstruction *μ*CT. (**d** and **e**) Formation of mineralized nodules in BMSCs from the above mice. BMSCs were isolated from the indicated mice and were cultured in the media containing ascorbic acid, *β*-glycerophosphate, and dexamethasone in the presence or absence of indicated concentration of tadalafil. After incubation for 21 days, cells were detected for bone nodules by alizarin-red staining and quantitative determination. (**f**–**i**) Tadalafil treatment reduced the mRNA levels of osteoblast marker genes (*AP* and *RunX2*) and target genes of canonical Wnt signaling (*Lef1* and *Dkk1*) in BMSCs from the femur and tibia of above mice. (**h**) Immunohistochemistry analyses of AP, Runx2, Lef1, and DKK1 expression in the distal femur of SFRP1^+/−^ or SFRP1^−/−^ mice treated with vehicle or indicated dosages of tadalafil. *P<0.05, **P<0.01 *versus* vehicle treatment.

We next assessed the effect of tadalafil on osteoblastogenesis and Wnt signaling in 2-month-old SFRP1 knockout (SFRP1^−/−^) mice. At the baseline level, SFRP1^−/−^ mice had a significantly higher mass of cancellous bone but not cortical bone as compared with SFRP1^+/−^ mice, as revealed by histological analysis of the longitudinal sections of the distal femur. At an oral dose of 75 mg/kg daily for 2 months, tadalafil robustly decreased the mass of cancellous bone but not in cortical bone in SFRP1^−/−^ mice (Figure 6a). Three-dimensional reconstruction of the distal femur using *μ*CT further confirmed that there were significant increases in BMD (2.1-fold), BMTV (2.3-fold), and TbN (1.5-fold) but not in TbTh and TbSp in SFRP1^−/−^ mice as compared with SFRP1^+/−^ mice, and that tadalafil treatment resulted in significant decreases in BMD (38%), BMTV (28%), and TbN (47%), but not in TbTh and TbSp as compared with the vehicle treatment (Figures 6a–c, and Supplementary Figure 4c and d). Likewise, BMSCs from SFRP1^−/−^ mice formed much more mineralized nodules than those from SFRP1^+/−^ mice (1.4-fold), and tadalafil attenuated SFRP1 knockout-associated excessive formation of mineralized nodules by ~25% over vehicle (Figures 6d and e). Moreover, BMSCs from SFRP1^−/−^ mice exhibited higher transcription activities of *Lef1 and Dkk1* (13- and 14-fold, respectively) than those from SFRP1^+/−^ mice, whereas systemic treatment with tadalafil in SFRP1^−/−^ mice robustly attenuated the transcription of these genes (39 and 54%, respectively; Figures 6h and i). Similarly, BMSCs from SFRP1^−/−^ mice also exhibited higher transcriptional activities of osteoblast differential markers including AP and Runx2 (24- and 26-fold, respectively) than those from SFRP1^+/−^ mice, and systemic treatment with tadalafil in SFRP1^−/−^ mice robustly attenuated the transcription of these markers (21 and 16%, respectively; Figures 6f and g). As demonstrated by immunohistochemistry staining of the sections of the distal femur, deletion of SFRP1 in mice led to a robust increase in the numbers of Lef1- and Dkk1-positive cells, as well as AP- and Runx2-positive cells, and that systemic treatment with tadalafil in SFRP1^−/−^ mice robustly attenuated the increases in the number of not only Lef1- and Dkk1-positive cells but also AP- and Runx2-positive cells (Figure 6j). As indicated by tartrate-resistant acid phosphatase (TRAP) staining, neither deletion of SFRP1 nor systemic treatment with tadalafil had any significant effect on the number of TRAP-positive cells in SFRP1^−/−^ mice (Supplementary Figure 3a and 3b). Together, these results further confirm that systemic inhibition of PDE5 specifically reduces the excessive bone growth derived from forced activation of canonical Wnt signaling in adult SFRP1^−/−^ mice.

## Discussion

**Figure 7:**
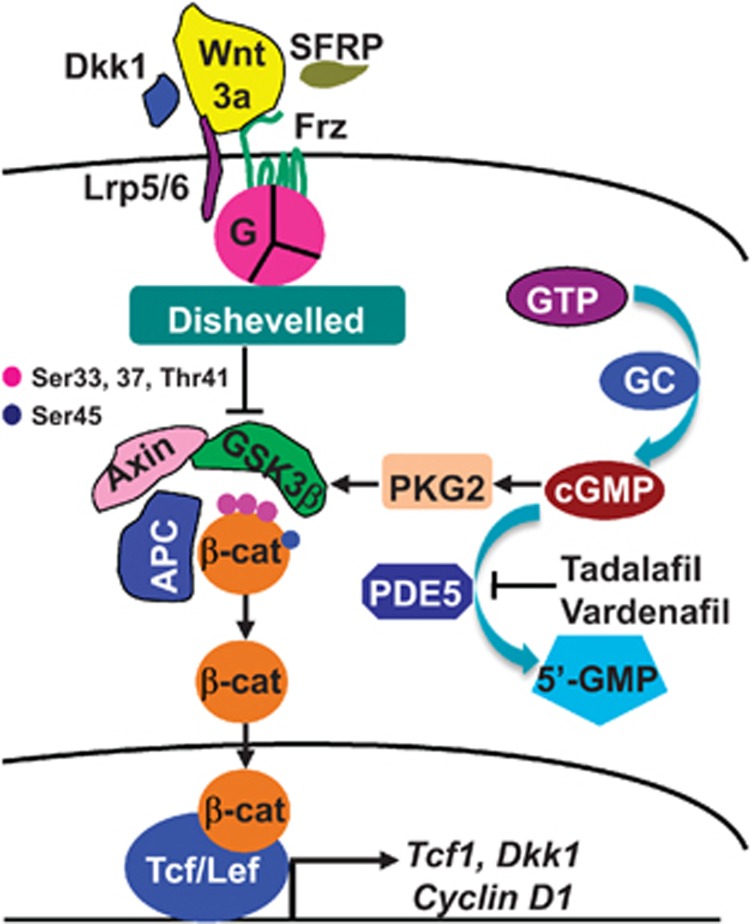
Schematic graph showing the proposed model for PDE5 inhibition-regulating canonical Wnt signaling and bone homeostasis. Inhibition of PDE5 by its inhibitors, such as tadalafil and vardenafil, results in elevation of cGMP and activation of cGMP-dependent protein kinase 2, which further activates GSK3*β* and thereby destabilizes *β*-catenin in the cytosol, leading to suppression of canonical Wnt signaling and the consequent reduction of osteoblastogenesis and bone mass. PDE5, phosphodiesterase 5; cGMP, cyclic guanosine monophosphate; GTP, guanosine-5′-triphosphate; GC, guanylyl cyclase; 5′-GMP, guanosine-5′-monophosphate; PKG, cGMP-dependent protein kinase; Dkk1, Dickkopf-1; SFRP, secreted Frz-related proteins; Frz, Frizzled; LRP5/6, low-density lipoprotein receptor-related protein 5 or 6; G, G protein; GSK3*β*, glycogen synthase kinase 3*β*; APC, adenomatous polyposis coli; *β*-cat, *β*-catenin; TCF, T-cell factors; Lef, lymphoid enhancer-binding factor

By using wild-type C57BL/6 and SFRP1 knockout mice, we have uncovered that the PDE/cGMP/PKG2 signaling operates in conjunction with GSK3*β*-mediated *β*-cat stabilization to regulate canonical Wnt signaling in maintenance of bone mass in adult mice *in vivo.* Using 293T and C3H10T1/2 cells, we have shown that inhibition of PDE5 induces cGMP-dependent PKG2, which activates GSK3*β* and thereby destabilizes *β*-cat in the cytosol, resulting in inhibition of canonical Wnt signaling and consequent reduction of osteoblastic differentiation *in vitro* (Figure 7).

In one of our previous studies, we have demonstrated that Wnt3a through LRP5/6, Dvl, and most likely Frz receptors, activates a signaling module composed of Gαq/11*βγ*-PI3K-Rac1-JNK2, resulting in stabilization of *β*-cat through phosphorylation at Ser191 and Ser605, and consequent localization to the nucleus.^16^ Here we show that PDE5 inhibition leads to activation of cGMP/PKG2, which destabilizes *β*-cat and consequently suppresses canonical Wnt signaling, supporting a role for the non-canonical pathway in the regulation of canonical Wnt signaling.^19^ The mechanisms underlying the activation of canonical *versus* non-canonical pathways by Wnt ligands are not fully understood.^11^ It has been suggested that the Frz2/Gαi/PDE5/cGMP/PKG signaling module is an important component of non-canonical Wnt signaling in the activation of intracellular Ca^2+^ transient.^15^ Here we report that PDE5/cGMP/PKG signals are also involved in canonical Wnt signaling. It seems that non-canonical Wnt signaling and canonical Wnt signaling operate interactively and the binary distinctions of these two Wnt pathways have come under scrutiny. As overexpression of Frz4 in HEK293 cells or Frz5 in *Xenopus* embryos is sufficient to transduce canonical signaling by Wnt5a,^20, 21^ the specificity of the underlying signaling complexes may be dictated by the Frz receptor(s).

A previous study has observed that activation of Frz2 induces the cGMP-dependent phosdiesterase and decreases the intracellular cGMP levels,^15^ suggesting that Wnt3a fails to activate Frz2, and that PDE5/cGMP/PKG signals regulate canonical Wnt signaling independently of Wnt3a. The current study has shown that PDE5/cGMP/PKG2 signals regulate the stabilization of *β*-cat at the protein but not the mRNA level. Inconsistent to this observation, Li *et al.*^22^ and Tinsley *et al.*^23^ have shown that both non-steroidal anti-inflammatory drug, sulindac sulfide (SS), and PDE5 inhibitor, tadalafil, inhibit PDE5 activity and increase cGMP levels, resulting in PKG activation, decreased proliferation, and apoptosis through transcriptional suppression of *β*-cat, but not its proteolytic degradation in colon cancer cells, and more recently, Tinsley *et al.*^24^ have demonstrated that SS may trigger *β*-cat protein degradation and suppress *β*-cat gene transcription through increased phosphorylation in human breast cancer cells. The inconsistency is possibly due to the use of different cell types in the previous studies (i.e., cancer cells) and this study (i.e., normal cells).

Inhibition of PDE5 and activation of cGMP/PKG2 induce the phosphorylation of *β*-cat at Thr41, Ser37, and Ser33 but not at Ser45, suggesting that GSK3*β* but not CK1 may be downstream of the PDE5/cGMP/PKG2 signaling module. This notion is further supported by the findings that knockdown of PKG2 is sufficient to activate the phosphorylation of GSK3*β* and the stabilization of *β*-cat. Finally, PKG2 and GSK3*β* co-existing within one immunoprecipitated complex prompts us to speculate that PKG2 directly phosphorylates GSK3*β* in the complex. In line with this speculation, several recent reports have shown physical interactions between PKG2 and GSK3*β*. In ATDC5 cells, PKG2 directly phosphorylates GSK3*β* to promote the hypertrophic differentiation.^25^ In UMR106 cells, PKG2 also phosphorylates the GSK3*β* at Ser9, and the apparent *Km* of PKG2 for GSK3*β* is about 0.15 xM.^26^ Thus, the present study has identified a mechanism in which activation of cGMP/PKG2 by inhibition of PDE5 results in GSK3*β*-mediated destabilization of *β*-cat and consequent suppression of canonical Wnt signaling.

The role for canonical Wnt signaling in the embryonic development of bone is well established, but the function in maintaining the bone homeostasis in adulthood is less clear.^27, 28^ With regard to non-canonical Wnt signaling, little is known regarding its role in bone development and homeostasis in both embryogenesis and adulthood.^11^ Consistent with the role of PDE5/cGMP/PKG2 signaling in Wnt3a-induced osteoblastogenesis *in vitro*, systemic administration of a PDE5 inhibitor, tadalafil, reduces bone mass not only in normal but also in SFRP1^−/−^ knockout adult mice in this study. The severe phenotype after tadalafil treatment reflects the importance of PDE5 activities in the maintenance of bone homeostasis. Endogenous Wnt signaling plays an important role in bone formation through stimulating the osteoblastogenesis and suppressing the adipogenesis and osteoclastogenesis.^29^ Our *in vivo* analyses show that: (1) inhibition of PDE5 reduces the trabecular bone but not the cortical bone, which is identical to the phenotypes of SFRP1 or LRP5 gain- and loss-of-function;^30, 31, 32^ (2) inhibition of PDE5 leads to the robust inhibition of *Lef1* and *Dkk1* transcription in BMSCs; (3) inhibition of PDE5 almost abolishes the SFRP1 knockout-producing overgrowth of bone; and (4) neither inhibition of PDE5 nor SFRP1 gain-of-function affects the osteoclastogenic activities. Together, these *in vivo* observations imply that inhibition of PDE5 attenuates not only the osteoblastogenic differentiation in response to Wnt3a but also the activities of canonical Wnt signaling, suggesting that inhibition of PDE5 reduces osteoblastogenesis possibly through suppressing canonical Wnt signaling.

Nitric oxide synthase (NOS) is the enzyme responsible for producing NO and NO activates soluble guanylyl cyclases to increase the intracellular cGMP levels and activate PKG.^14^ Consistent with our findings, previous studies have demonstrated that mice lacking endothelial NOS exhibit profound abnormalities in bone formation and a significant delay in osteoblastic differentiation,^33, 34^ and that mice lacking either PKG2 or PKG1 may develop severe phenotypes; however, analyses of the skeletons of PKG2 but not PKG1 mutants have revealed the obvious defects in chondrogenesis but not in osteoblastogenesis.^25, 35, 36, 37, 38, 39^ It is likely that PKG2 and PKG1 play a functional redundancy in osteoblastogenesis.

In summary, the present study has demonstrated that PDE5 inhibition may cause bone mass loss involving non-canonical Wnt pathway, which operates interactively with canonical Wnt signaling in a way to destabilize *β*-cat through a cGMP/PKG2/GSK3*β* signaling-dependent mechanism. Given that PDE5 inhibitors are commonly used in patients with ED and PH, our findings may have significant clinical implications in alerting physicians of putative adverse effect of PDE5 inhibitors.

## Materials and Methods

### Chemicals and antibodies

Tadalafil and vardenafil were obtained from Selleckchem (Houston, TX, USA). KT5823, 8-Br(Bromo)-cGMP, Alizarin Red S, and SB216763 were from Sigma (St Louis, MO, USA). Recombinant murine Wnt3a protein was purchased from R&D System (Minneapolis, MN, USA). Antibodies against PKG1, PKG2, Lamin B, Dkk1, IgG, *β*-actin, and protein A/G PLUS-Agarose were from Santa Cruz Biotechnology (Santa Cruz, CA, USA). Antibodies against p-*β*-cat (Ser33/37/Thr41), p-*β*-cat (Ser45), *β*-cat, p-GSK3*β* (Ser9) and GSK3*β* were from Cell Signaling (Danvers, MA, USA). Antibodies against AP and RunX2 were purchased from Abcam (Cambridge, UK), and the antibody against Lef1 was from Proteintech (Chicago, IL, USA). The IRDye 680 and 800 second antibodies were from LI-COR Bioscience (Lincoln, Nebraska) and the Alexa 555-conjugated secondary antibodies were from Life Technologies (Grand Island, NY, USA). Cyclic GMP Direct Immunoassay kits were obtained from Abcam and TRAP staining kits were from Sigma.

### Cell culture and conditioned medium preparation

C3H10T1/2 cells, Wnt3a-expressing and control L cells, and HEK293T cells were all obtained from ATCC (Manassas, VA, USA). For maintenance, C3H10T1/2 cells and HEK293T cells were cultured, respectively, in Basal Medium Eagle (Life Technologies) and Dulbecco's modified Eagle's medium (Life Technologies) supplemented with 10% fetal calf serum (FCS, Life Technologies). Primary bone marrow stromal cells (BMSCs) were isolated from the femur and tibia, and cultured in alpha Minimum Essential Media (Life Technologies) with 15% FCS as described previously.^11^ Conditional media were prepared from Wnt3a-expressing and control L cells, respectively, as described previously.^16^

### Transient transfection and dual-luciferase assay

SV40 large T-antigen-expressing HEK cells (293T cells) were plated onto 24-well plates. Next day, they were transfected with 2 *μ*g Lef1 reporter construct using Lipofectamine 2000 reagent and 0.02 *μ*g Renilla luciferase construct (Promega, Madison, WI, USA) for 6 h in the absence of serum.^40^ In some cases, the cells were co-transfected with siRNA oligonucleotides targeting the test genes *PDE5a*, *PKG1*, *PKG2*, and *GSK3β* (see Supplementary Table 1 for the specific sequence information), Lef1 reporter construct and Renilla construct. Transfectants were then cultured in a 1 : 2 diluted conditioned medium prepared from control L cells or Wnt3a-expressing cells in the presence of tadalafil and/or vardenafil for 48 h. At the end of the designated culture, cell lysates were prepared and dual-luciferase assay was performed according to the manufacturer's instructions (Promega). The firefly luciferase activity was normalized to the Renilla luciferase activity.

### Western blotting, immunoprecipitation and immunocytochemistry

293T and C3H10T1/2 cells were plated 1 × 10^4^ and 1.5 × 10^4^ cells/cm^2^ overnight, after a variety of treatments. Cytosolic and nuclear fractions of cells were prepared by using NE-PER Nuclear and Cytoplasmic Extraction Reagents (Thermo Scientific, Waltham, MA, USA) as per the manufacturer's instructions. *β*-Actin and Lamin-B were used as the internal standards for the cytosolic and nuclear fractions, respectively. Western blot analysis and immunoprecipitation were performed using standard protocols and the immunoreactive signals for the proteins of interest were quantitated using the LI-COR Odyssey Infrared Imaging System (LI-COR Bioscience) and ImageJ (http://rsb.info.nih.gov/ij/). 293T cells seeded on chamber slides (Nalge Nunc International, Rochester, NY, USA) at 0.75 × 10^4^/cm^2^ and cultured in regular medium overnight and in serum-free medium for 12 h. After pre-treatment with tadalafil for 2 h, serum-starved cells were further stimulated with recombinant murine Wnt3a protein at 100 ng/ml in fresh serum-free medium for 30 min. Cells were then immunostained with primary antibodies against *β*-catenin and subsequently counterstained with 4',6-diamidino-2-phenylindole. Immunoreactive protein signals were examined by confocal microscopy.

### Osteoblast differentiation assay and quantitative RT-PCR

C3H10T1/2 cells were seeded onto six-well plates. At confluence, cells were stimulated with conditioned medium prepared from control L or Wnt3a-expressing cells for 48 h in the presence of vehicle or tadalafil. AP activity was determined and expressed as nanomoles of p-nitrophenol formed per minute per milligram of protein as previously described.^41^. For mineralization assays, confluent cells were incubated in the presence of 50 mg/ml ascorbic acid and 50 mM *β*-glycerophosphate for 21 days. Total RNA was extracted using TRIzol reagent (Takara Biotechnology (Dalian) Co., Ltd., Dalian, China). mRNA levels of osteoblast markers and target genes of canonical Wnt signaling including *Dkk1*, *Lef1*, *AP*, *Runx2*, and *Osx* were determined by real-time PCR as previously described.^42, 43^ GAPDH was included as an internal control and the relative levels of mRNA species of interest were calculated by the 2^−ΔΔCt^ method.^44^ The primers used in our PCR analysis are presented in the Supplementary Information (Supplementary Table 2).

### Mouse strains, treatments and histological assessment

Male C57BL/6 mice were purchased from Shanghai SLAC Laboratory Animal Co. (Shanghai, China) and founder SPRP1^+/−^ mouse strains were gifted by Dr Akihiko Shimono.^45^ Two-month-old mice were orally administrated with tadalafil daily at 0, 45 or 75 mg/kg for 2 months. We used *μ*CT (*μ*CT 40, Scanco Medical AG, Brüttisellen, Switzerland) for three-dimensional reconstruction and quantification of bone parameters, and reconstructed each image from one hundred 16-*μ*m slices immediately below the growth plate, with a threshold of 200.^46^ Histological examination and immunohistochemistry staining were performed on paraffin sections (4 *μ*m) after decalcification. Immunohistochemistry staining was performed by using the Histostain-Plus Kit (Kangwei Reagents, Beijing, China) as described previously.^47^ After sequential treatments, tissue sections were sequentially incubated with normal serum for 30 min, control IgG and primary antibodies against AP, Runx2, Lef1, or Dkk1 for 2 h, and then HRP-conjugated secondary antibody for 30 min. The diaminobenzidine solution was used for development of brown color and sections were counterstained with hematoxylin. The quantitative histomorphometry was performed using the Osteomeasure Analysis System (OsteoMetrics, Inc., Decatur, GA, USA). The Animal Studies Committee at Zhejiang University approved all mouse procedures.

### Statistical analysis

Experiments were repeated at least three times. Numerical data were expressed as means±S.D. and analyzed by one-way ANOVA and Tukey–Kramer multiple comparisons test. Differences were considered significant when *P*<0.05. The SPSS statistical package (IBM, North Castle, NY, USA) was used.

## Abbreviations

AP: alkaline phosphatase
*β*-cat: *β*-catenin
BMSC: bone marrow-derived stromal cell
BMTV: trabecular bone volume
cGMP: cyclic guanosine monophosphate
CK: casein kinase
Dkk1: Dickkopf-1
ED: erectile dysfunction
Frz: Frizzled
GSK3*β*: glycogen synthase kinase 3*β*
Lef: lymphoid enhancer-binding factor
LRP5/6: low-density lipoprotein receptor-related protein 5 or 6
NOS: nitric oxide synthase
OSX: ostrix
PDE5: phosphodiesterase 5
PH: pulmonary hypertension
PKG: cGMP-dependent protein kinase
Runx2: Runt-related transcription factor 2
SFRP: secreted Frz-related protein
SS: sulindac sulfide
TbN: trabecular number
TbSp: trabecular separation
TbTh: trabecular thickness
