# Supplementary material for: Inhibition of phosphodiesterase 5 reduces bone mass by suppression of canonical Wnt signaling
Source: Cell Death Dis. 2014 Nov 27;5(11):e1544–. doi: 10.1038/cddis.2014.510 (PMC4260761; doi:10.1038/cddis.2014.510)
Supplement: Supplementary Figures [file cddis2014510x1.ppt]

## Slide 1
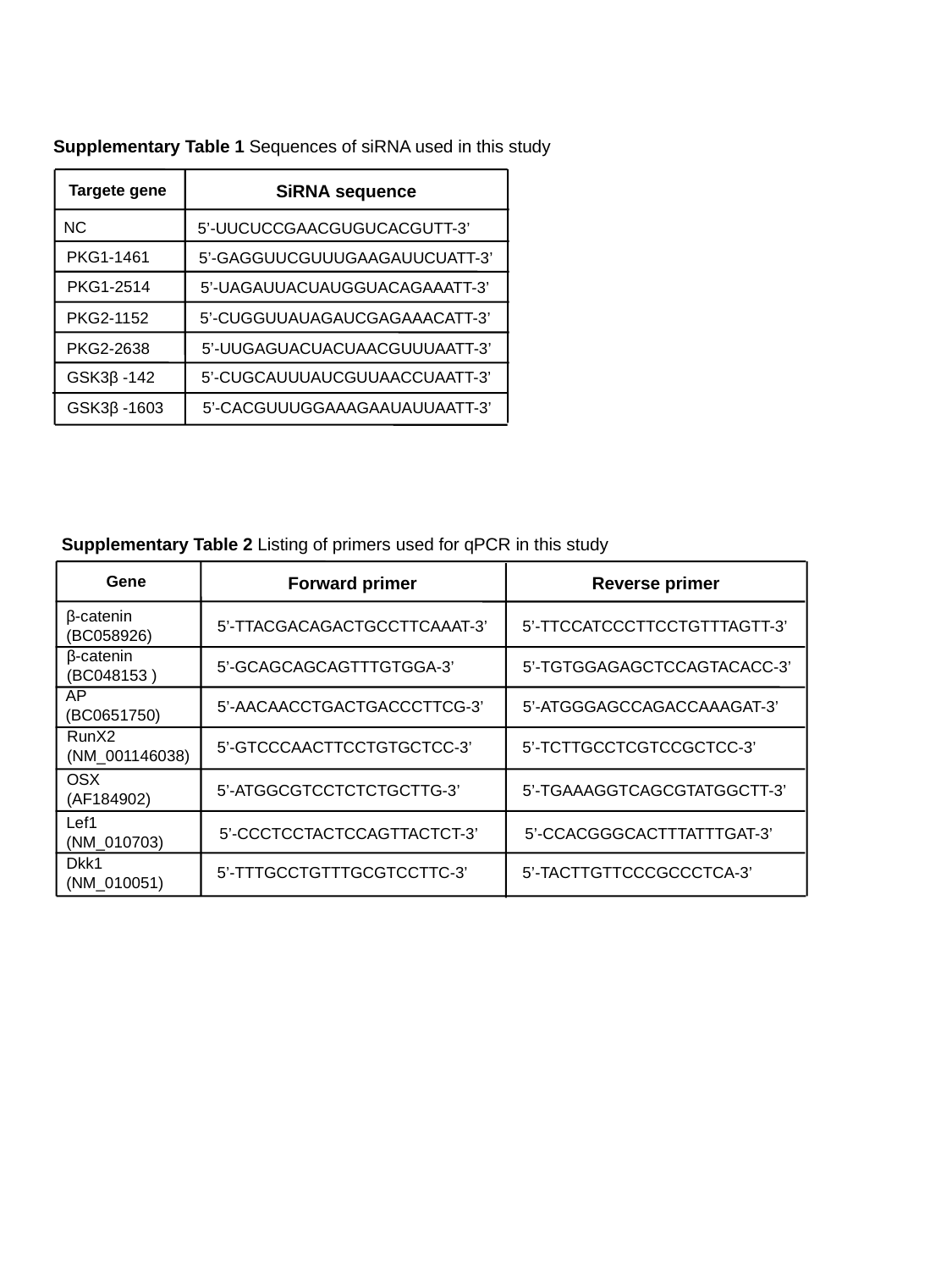

Supplementary Table 1 Sequences of siRNA used in this study
SiRNA sequence
Targete gene
NC
5’-UUCUCCGAACGUGUCACGUTT-3’
PKG1-1461
5’-GAGGUUCGUUUGAAGAUUCUATT-3’
PKG1-2514
5’-UAGAUUACUAUGGUACAGAAATT-3’
PKG2-1152
5’-CUGGUUAUAGAUCGAGAAACATT-3’
PKG2-2638
5’-UUGAGUACUACUAACGUUUAATT-3’
GSK3β -142
5’-CUGCAUUUAUCGUUAACCUAATT-3’
GSK3β -1603
5’-CACGUUUGGAAAGAAUAUUAATT-3’
Supplementary Table 2 Listing of primers used for qPCR in this study
Gene
Forward primer
Reverse primer
β-catenin
(BC058926)
5’-TTACGACAGACTGCCTTCAAAT-3’
5’-TTCCATCCCTTCCTGTTTAGTT-3’
β-catenin
(BC048153 )
5’-GCAGCAGCAGTTTGTGGA-3’
5’-TGTGGAGAGCTCCAGTACACC-3’
AP
(BC0651750)
5’-AACAACCTGACTGACCCTTCG-3’
5’-ATGGGAGCCAGACCAAAGAT-3’
RunX2
(NM_001146038)
5’-GTCCCAACTTCCTGTGCTCC-3’
5’-TCTTGCCTCGTCCGCTCC-3’
OSX
(AF184902)
5’-ATGGCGTCCTCTCTGCTTG-3’
5’-TGAAAGGTCAGCGTATGGCTT-3’
Lef1
(NM_010703)
5’-CCCTCCTACTCCAGTTACTCT-3’
5’-CCACGGGCACTTTATTTGAT-3’
Dkk1
(NM_010051)
5’-TTTGCCTGTTTGCGTCCTTC-3’
5’-TACTTGTTCCCGCCCTCA-3’
