# Supplementary material for: Inhibition of phosphodiesterase 5 reduces bone mass by suppression of canonical Wnt signaling
Source: Cell Death Dis. 2014 Nov 27;5(11):e1544–. doi: 10.1038/cddis.2014.510 (PMC4260761; doi:10.1038/cddis.2014.510)
Supplement: Supplementary Tables [file cddis2014510x2.ppt]

## Slide 1
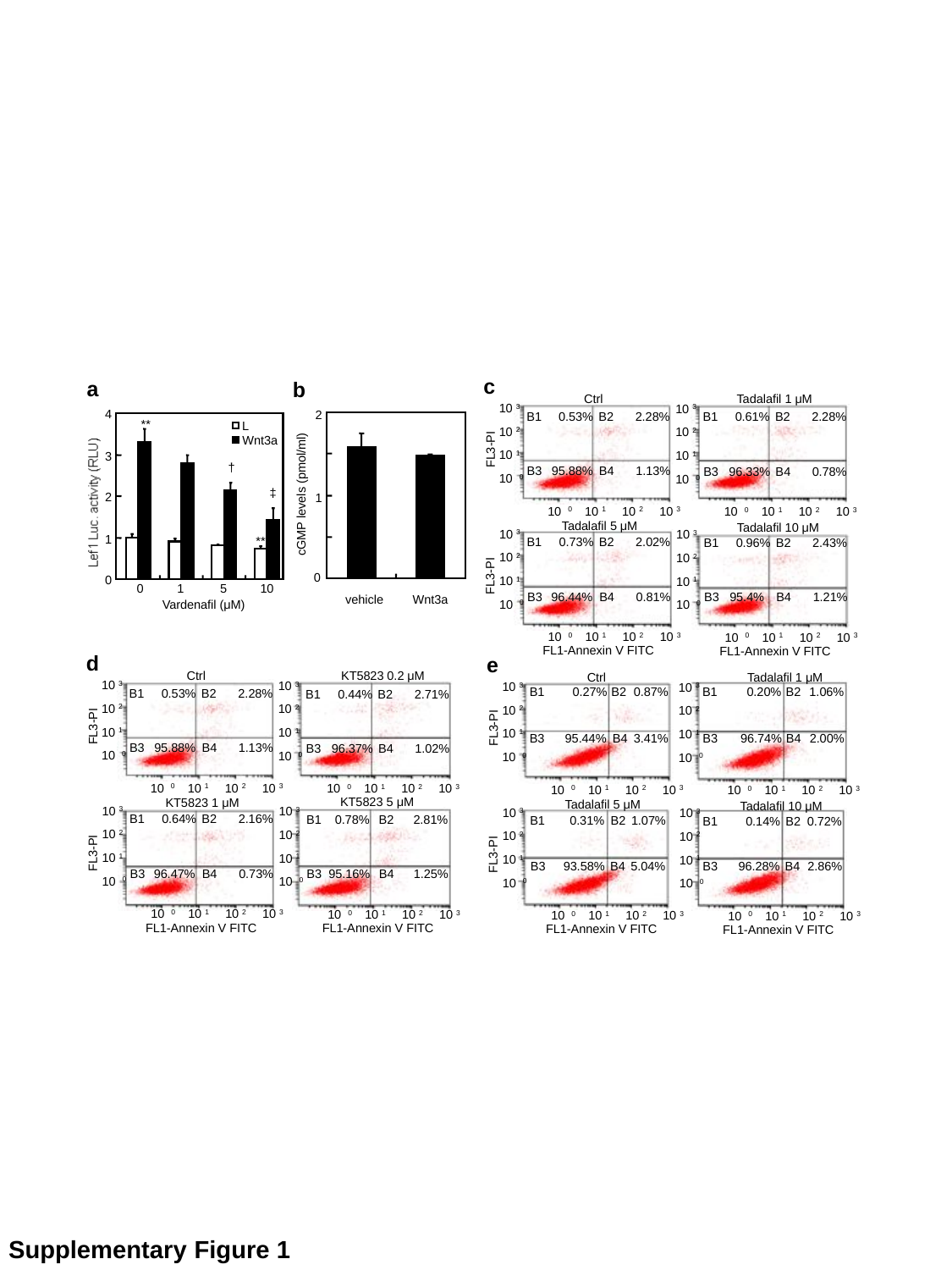

c
Ctrl
Tadalafil 1 μM
10 3
B1
0.53%
B2
2.28%
10 2
FL3-PI
10 1
B3
95.88%
B4
1.13%
10 0
10 0
10 1
10 2
10 3
10 3
B1
0.61%
B2
2.28%
10 2
10 1
B3
96.33%
B4
0.78%
10 0
10 0
10 1
10 2
10 3
Tadalafil 5 μM
Tadalafil 10 μM
10 3
B1
0.73%
B2
2.02%
10 2
10 1
B3
96.44%
B4
0.81%
10 0
10 0
10 1
10 2
10 3
FL3-PI
10 3
B1
0.96%
B2
2.43%
10 2
10 1
B3
95.4%
B4
1.21%
10 0
10 0
10 1
10 2
10 3
FL1-Annexin V FITC
FL1-Annexin V FITC
a
b
2
cGMP levels (pmol/ml)
1
0
vehicle
Wnt3a
4
**
L
Wnt3a
3
†
‡
2
**
1
0
0
1
5
10
Vardenafil (μM)
d
Ctrl
KT5823 0.2 μM
10 3
10 3
B1
0.53%
B2
2.28%
B1
0.44%
B2
2.71%
10 2
10 2
FL3-PI
10 1
10 1
B3
95.88%
B4
1.13%
B3
96.37%
B4
1.02%
10 0
10 0
10 0
10 1
10 2
10 3
10 0
10 1
10 2
10 3
KT5823 5 μM
KT5823 1 μM
10 3
B1
0.64%
B2
2.16%
10 2
10 1
B3
96.47%
B4
0.73%
10 0
10 0
10 1
10 2
10 3
10 3
B1
0.78%
B2
2.81%
10 2
FL3-PI
10 1
B3
95.16%
B4
1.25%
10 0
10 0
10 1
10 2
10 3
FL1-Annexin V FITC
FL1-Annexin V FITC
e
Ctrl
Tadalafil 1 μM
10 3
10 3
B1
0.27%
B2
0.87%
B1
0.20%
B2
1.06%
10 2
10 2
FL3-PI
10 1
10 1
B3
95.44%
B4
3.41%
B3
96.74%
B4
2.00%
10 0
10 0
10 0
10 1
10 2
10 3
10 0
10 1
10 2
10 3
Tadalafil 5 μM
Tadalafil 10 μM
10 3
10 3
B1
0.31%
B2
1.07%
B1
0.14%
B2
0.72%
10 2
10 2
FL3-PI
10 1
10 1
B3
93.58%
B4
5.04%
B3
96.28%
B4
2.86%
10 0
10 0
10 0
10 1
10 2
10 3
10 0
10 1
10 2
10 3
FL1-Annexin V FITC
FL1-Annexin V FITC
Supplementary Figure 1

## Slide 2
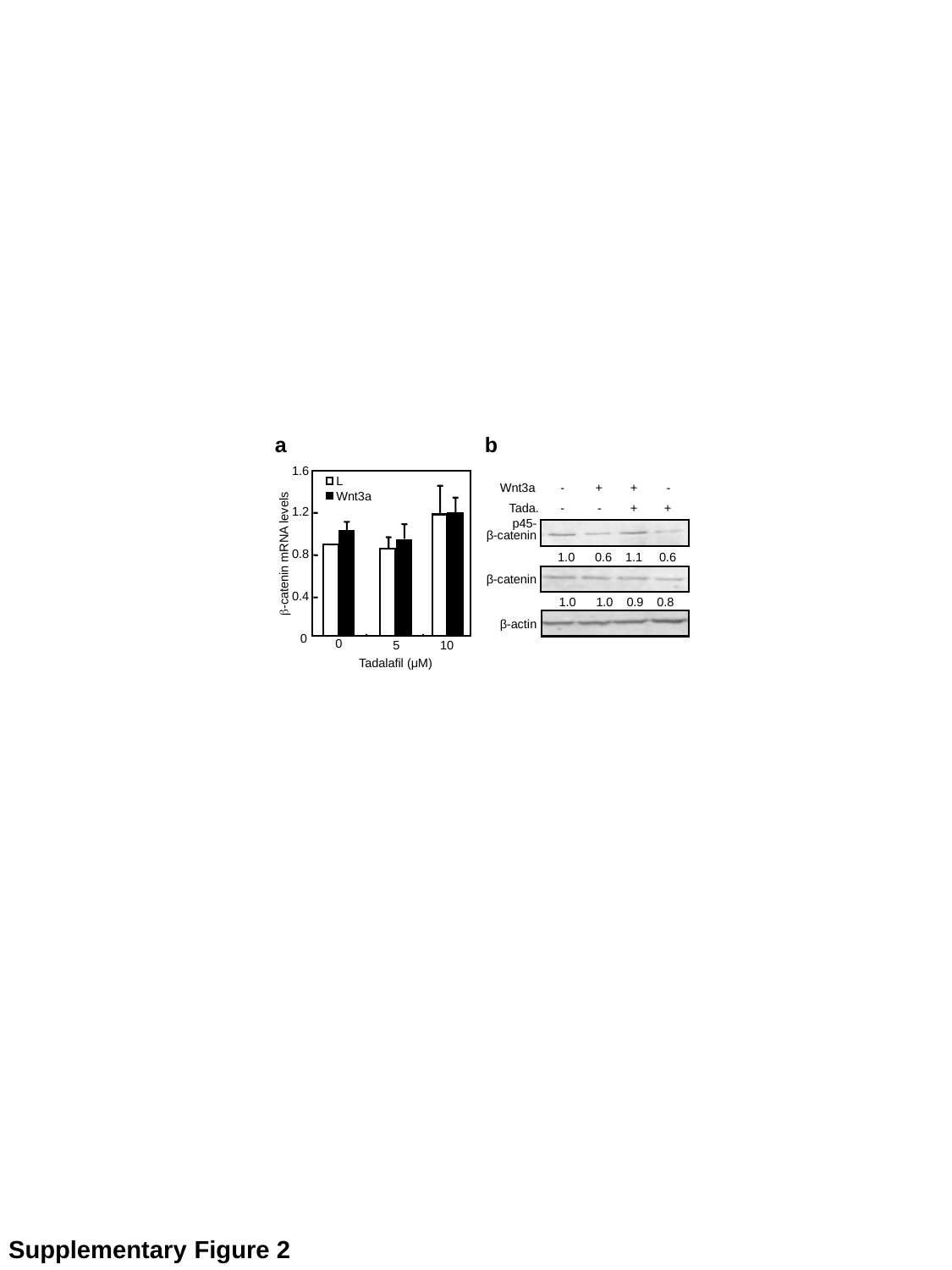

b
a
1.6
L
Wnt3a
-
+
+
-
Wnt3a
Tada.
-
-
+
+
1.2
p45-
β-catenin
β-catenin mRNA levels
1.0 0.6 1.1 0.6
0.8
β-catenin
1.0 1.0 0.9 0.8
0.4
β-actin
0
0
5
10
Tadalafil (μM)
Supplementary Figure 2

## Slide 3
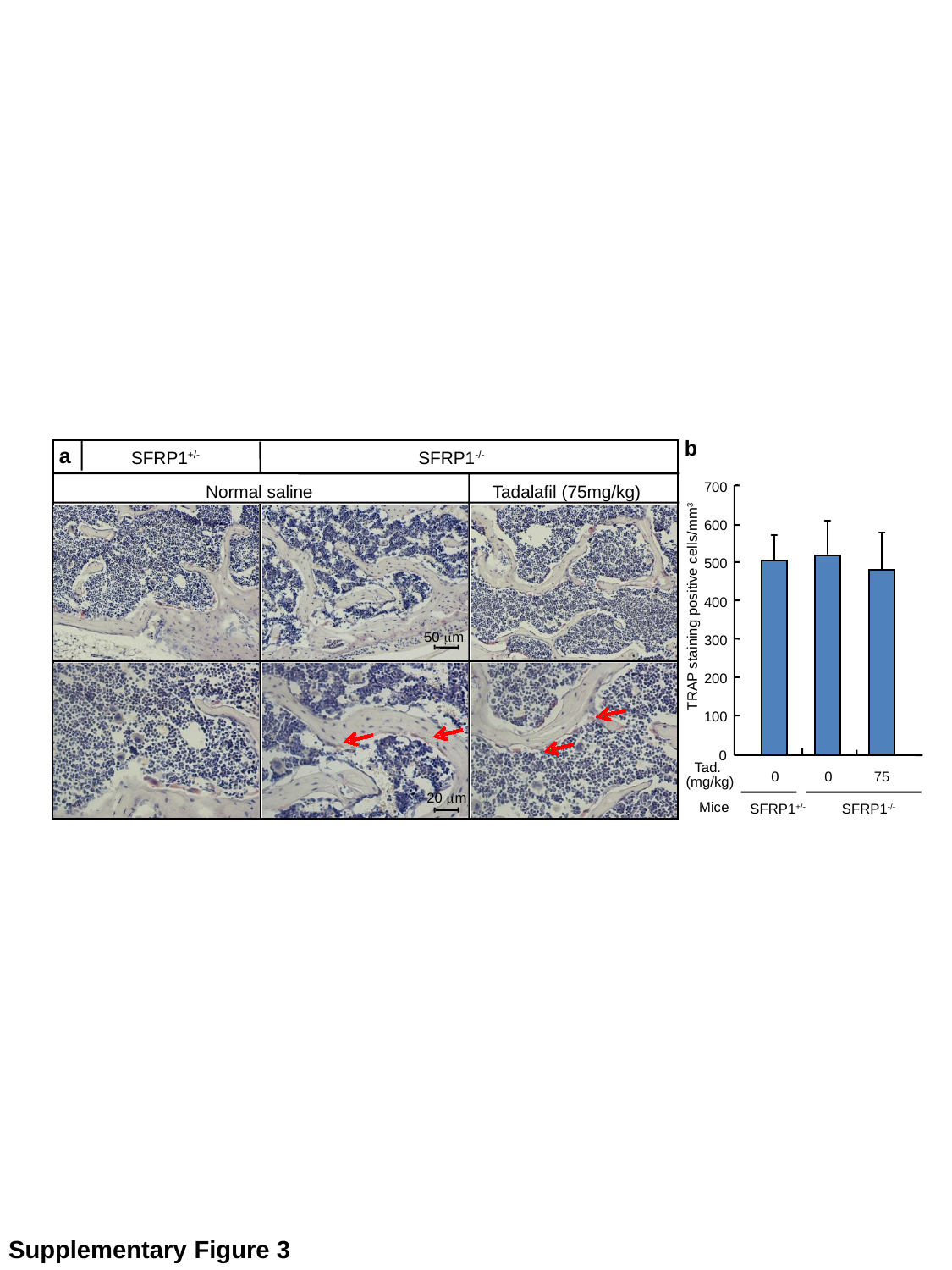

b
a
SFRP1+/- SFRP1-/-
Normal saline Tadalafil (75mg/kg)
700
600
500
TRAP staining positive cells/mm3
400
50 μm
300
200
100
0
Tad.
(mg/kg)
0
0
75
20 μm
Mice
SFRP1+/- SFRP1-/-
Supplementary Figure 3

## Slide 4
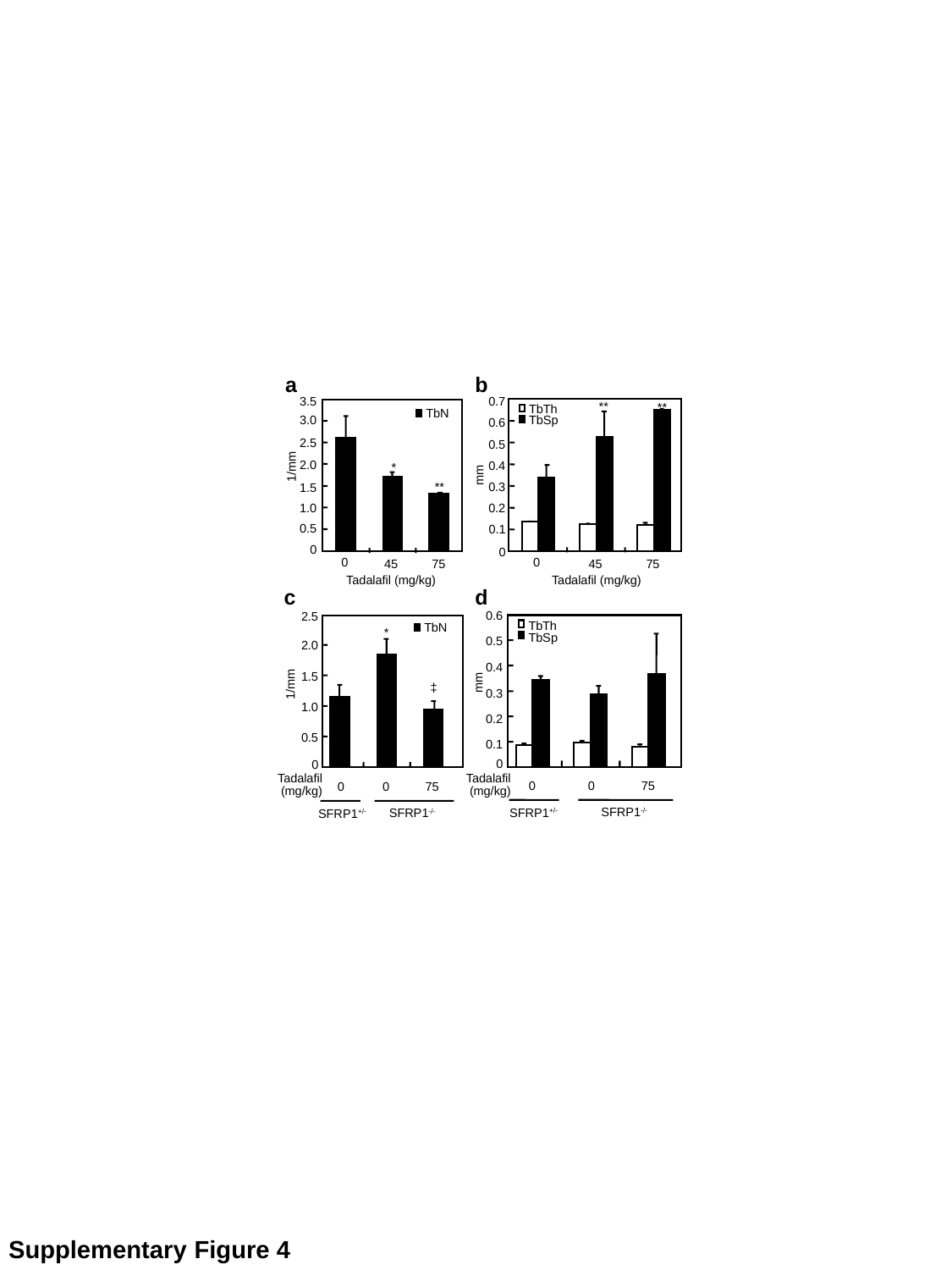

a
b
**
**
3.5
0.7
TbTh
TbN
TbSp
3.0
0.6
2.5
0.5
1/mm
*
2.0
0.4
mm
**
0.3
1.5
1.0
0.2
0.5
0.1
0
0
0
0
45
45
75
75
Tadalafil (mg/kg)
Tadalafil (mg/kg)
c
d
0.6
2.5
*
TbTh
TbSp
TbN
0.5
2.0
0.4
mm
1.5
1/mm
‡
0.3
1.0
0.2
0.5
0.1
0
0
Tadalafil
(mg/kg)
Tadalafil
(mg/kg)
0
0
75
0
0
75
SFRP1-/-
SFRP1+/-
SFRP1-/-
SFRP1+/-
Supplementary Figure 4
